# Expression of TRAF6 in peripheral blood B cells of patients with myasthenia gravis

**DOI:** 10.1186/s12883-022-02833-9

**Published:** 2022-08-17

**Authors:** Ting Li, Yue Li, Jia-Wen Li, Ying-Hui Qin, Hui Zhai, Bin Feng, He Li, Ning-Nannan Zhang, Chun-Sheng Yang

**Affiliations:** 1 Department of Neurology, Tianjin Neurological Institute, Tianjin Medical University General Hospital, No. 154 Anshan Road, Heping District, Tianjin, 300052 China; 2 Department of Neurology, Tianjin Huanhu Hospital, Tianjin, China; 3 Department of Radiology and Tianjin Key Laboratory of Functional Imaging, School of Medical Imaging, Tianjin Medical University General Hospital, Tianjin Medical University, No. 154 Anshan Road, Heping District, Tianjin, 300052 China

**Keywords:** Myasthenia gravis, Tumor necrosis factor receptor (TNFR)-associated factor 6 (TRAF6), B cell, Memory B cell, ADL-score

## Abstract

**Background:**

Tumor necrosis factor receptor-associated factor 6 (TRAF6) can regulate the activation of inflammatory signaling pathways by acting as an E3 ubiquitin ligase, which enhances B cell activation. This study aimed to evaluate the expression of TRAF6 in the peripheral blood B cells of myasthenia gravis (MG) patients and analyze the relationships between TRAF6 expression and clinical characteristics.

**Method:**

In our study, the expression level of TRAF6 in peripheral blood B cells of 89 patients was measured by flow cytometry compared with that of healthy subjects. The effects of disease severity, MG classification and immunotherapy on TRAF6 expression level were also analyzed.

**Results:**

In our study, TRAF6 expression was elevated in CD19^+^ B cells and CD19^+^CD27^+^ memory B cells in generalized MG (GMG) patients compared with ocular MG (OMG) patients (*p* = 0.03 and *p* = 0.03, respectively). There was a significant positive correlation between the TRAF6 expression level and disease severity in both OMG patients and GMG patients (CD19^+^ B cells: OMG: *p* < 0.001, *r* = 0.89; GMG: *p* = 0.001, *r* = 0.59; CD29^+^CD27^+^ B cells: OMG: *p* = 0.001, *r* = 0.80; GMG: *p* = 0.048, *r* = 0.38). TRAF6 expression was significantly elevated in CD19^+^ B cells and CD19^+^CD27^+^ memory B cells in GMG with acute aggravation compared with GMG in MMS (*p* = 0.009 and *p* = 0.028, respectively). In the eleven MG patients who were followed, TRAF6 expression in B cells and memory B cells was significantly decreased after treatment (*p* = 0.03 and *p* < 0.01, respectively).

**Conclusion:**

TRAF6 is potentially a useful biomarker of inflammation in patients with MG, and might be used to evaluate the effectiveness of treatment.

**Supplementary Information:**

The online version contains supplementary material available at 10.1186/s12883-022-02833-9.

## Introduction

Myasthenia gravis (MG) is a prototypical B cell-mediated autoimmune disease that mainly affects the postsynaptic membrane at the neuromuscular junction (NMJ) [1, 2]. In eighty percent of patients, MG is caused by autoantibodies against the postsynaptic acetylcholine receptor (AChR), whereas in a small minority of patients, MG is caused by autoantibodies against muscle-specific kinase (MuSK) or lipoprotein-receptor-related protein 4 (LRP4) [1]. Abnormal B cell activation and autoantibody overproduction play crucial roles in the pathogenesis of MG.

Tumor necrosis factor receptor (TNFR)-associated factor 6 (TRAF6) is a member of the TRAF family that can regulate signaling pathways by acting as an E3 ubiquitin ligase in addition to its role as an adaptor protein [3, 4]. TRAF6 acts downstream of CD40 (a member of the TNFR superfamily) and the Toll-like receptor (TLR) superfamily to regulate the activation of the nuclear factor-kappaB (NF-κB) and mitogen-activated protein kinase (MAPK) signaling pathways [4–7], which enhances the activation of B cells and the expression of interleukin (IL)-6 [8], IL-4, IL-1 [9] and other downstream factors. Recently, TRAF6 expression was found to be upregulated in systemic lupus erythematosus (SLE), rheumatoid arthritis (RA), hyperplastic thymuses and MG patient serum. Studies have demonstrated that miR-146a and TRAF6 exhibit high mRNA expression in peripheral blood mononuclear cells (PBMCs) in MG patients [8, 10–12]. In addition, the relationships between TRAF6 expression in peripheral blood B cells and the clinical characteristics of MG patients have rarely been elucidated.

This study aimed to evaluate the expression of TRAF6 in the peripheral blood B cells of MG patients and analyze the relationships between TRAF6 expression and clinical characteristics.

## Materials and methods

### Patients

A total of 89 MG patients admitted to Tianjin Medical University General Hospital were recruited for this study from November 2020 to October 2021. Forty-two patients with acute aggravation of MG were enrolled before immunotherapy. Immunotherapy refers to steroids, immunoglobulins, immunosuppressants. In addition, we followed eleven general MG patients with acute aggravation before immunotherapy and collected blood samples again when they reached the MMS after treatment. Fifty-eight MG patients were enrolled who achieved the minimal manifestation state (MMS) after treatment, including 47 patients with MMS at enrollment and 11 patients who achieved MMS at follow-up. We enrolled 43 age and sex matched healthy controls (HCs) from the Tianjin Medical University General Hospital Healthy Care Center. All the healthy controls had no heart, digestive system, respiratory system, blood system or other nervous system diseases. MG was diagnosed from clinical symptoms, the effect of cholinesterase inhibitors, autoantibody testing, computed tomography (CT) scans and electromyography [13]. Both MG patients and HCs with other autoimmune diseases were excluded from this study. The disease severity of MG was evaluated with the Myasthenia Gravis Activities of Daily Living (MG-ADL) score.

The study was approved by the Tianjin Medical University General Hospital Institutional Review Board and Ethics Committee (Tianjin, China), and all the participants signed informed consent forms.

### PBMC preparation

Five milliliters of blood was collected from each subject in ethylenediaminetetraacetic acid (EDTA)-coated vacutainer tubes. PBMCs were isolated from the blood by Ficoll gradient centrifugation and incubated at 37 °C in 5% CO2 for 4 h with Cell Stimulation Cocktail (a premixed cocktail with optimized concentrations of phorbol 12-myristate-13-acetate (PMA), ionomycin and the protein transport inhibitor brefeldin A).

### Flow cytometry

PBMCs were washed with phosphate-buffered saline (PBS) and incubated with Fc Receptor Blocking reagent (BioLegend, San Diego, California, USA) for 10 min at room temperature. After washing, the PBMCs were surface stained with allophycocyanin (APC)-conjugated mouse anti-human CD19 (BioLegend, San Diego, California, USA) and PE-labeled mouse anti-human CD27 (BioLegend, San Diego, California, USA) antibodies. The PBMCs were intracellularly stained with Alexa Fluor 488-conjugated mouse anti-human TRAF-6 antibodies (R&D Systems, Minneapolis, MN, USA) after fixation and permeabilization. Isotype controls were used to ensure antibody specificity. We used the % positive ratio to analyze TRAF6 expression in B cells, and this ratio was calculated as the percentage of TRAF6^+^CD19^+^ B cells in the total CD19^+^ B cell population. Similarly, the % positive ratio of CD19^+^CD27^+^ memory B cells was calculated as the percentage of TRAF6^+^CD19^+^CD27^+^ memory B cells in the total CD19^+^CD27^+^ memory B cell population, and the data were analyzed using FlowJo software VX.

### Statistical analysis

SPSS 22.0 statistics program was used for statistical analysis, and graphs were generated using GraphPad Prism 8.1. Through the Shapiro–Wilk test of the data, we find that the data is not normally distributed. All the data are expressed as the median (P25, P75). Comparisons between HCs and MG patients or between ocular MG (OMG) and generalized MG (GMG) patients were conducted using the Mann–Whitney U test. Kruskal–wallis method were used to compare the differences among the three groups, and Dunn method was used to correct *p* values. Spearman’s correlation coefficients were used to analyze the correlation between the TRAF6-positive ratio and ADL score. *P* values less than 0.05 were considered statistically significant.

## Results

### General clinical characteristics

The baseline characteristics of the patients with MG are shown in Table 1.

**Table 1:** General clinical characteristics

	MG (*n* = 89)	HC (*n* = 43)	*P* value
Age (mean ± SD)	58.34 ± 14.99	55.72 ± 19.51	0.94
Sex (male/female)	44/45	27/16	0.18
Onset age	55.36 ± 15.20		
EOMG	28		
LOMG	61		
AChR-Ab ( +)	81		
MuSK-Ab ( +)	1		
AChR-Ab/MuSK-Ab (-)	7		
OMG	38		
GMG	51		
Thymoma	22		
Thymic hyperplasia	3		
Acute aggravation state	42		
OMG	14		
GMG	28		
MGFA-I	14		
MGFA-II	10		
MGFA-III	14		
MGFA-IV	3		
MGFA-V	0		
MMS^a^	58		
Immunotherapy			
Tacrolimus only	13		
Corticosteroid only	19		
Azathioprine only	1		
Cyclophosphamide only	1		
Tacrolimus and corticosteroid	11		
Azathioprine and corticosteroid	2		

*Abbreviations: MG* Myasthenia gravis, *HC* Healthy control, *n* Number, *EMOG* Early-onset myasthenia gravis, *LOMG* Late-onset myasthenia gravis, *AChR-Ab* Acetylcholine receptor-specific antibody, *MuSK-Ab* Muscle-specific kinase-specific antibody, *OMG* Ocular myasthenia gravis, *GMG* Generalized myasthenia gravis, *MGFA* MG Foundation of America, *MMS* Minimal manifestation status

^a^MMS: fifty-eight MG patients were enrolled who achieved the minimal manifestation state (MMS) after treatment, including 47 patients with MMS at enrollment and 11 patients who achieved MMS at follow-up

### Expression of TRAF6 in MG patients

**Fig. 1:**
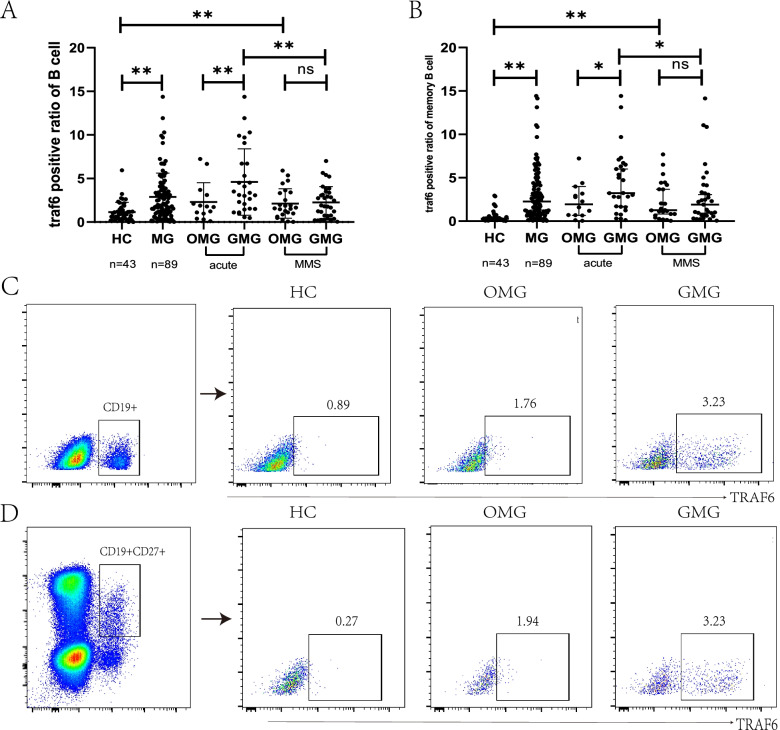
**A** Comparison of the TRAF6-positive ratio in CD19^+^ B cells among different groups (HCs: 0.89 (0.36, 1.45), MG: 1.94 (1.018, 3.773), *p* < 0.01; acute aggravation state: OMG: 1.76 (0.83, 3.26), GMG: 3.23 (1.51, 6.70), *p* = 0.03). Acute refers MG patients with acute aggravation state but before any immunotherapy. TRAF6 expression was significantly elevated in CD19^+^ B cells and CD19^+^ CD27^+^ memory B cells in GMG with acute aggravation compared with GMG in MMS (*p* = 0.009, *p* = 0.028 respectively). MMS patients were those who had achieved a minimal manifestation state (MMS) after immunotherapy or nonimmunotherapy. **B** Comparison of the TRAF6-positive ratio in CD19^+^CD27^+^ B cells among different groups (HCs: 0.27 (0.16, 1.57), MG: 2.25 (0.77, 4.42), *p* < 0.01; acute aggravation state: OMG: 1.94 (0.68, 3.38), GMG: 3.23 (1.61, 6.45), *p* = 0.03). **C** Flow cytometry analysis of TRAF6 in CD19^+^ B cells from healthy controls (HCs), ocular myasthenia gravis (OMG) patients and generalized myasthenia gravis (GMG) patients with acute aggravation. **D** Flow cytometry analysis of TRAF6 in CD19^+^CD27^+^ memory B cells from HCs, OMG patients and GMG patients with acute aggravation

In general, the TRAF6 expression levels in CD19^+^ B cells and CD19^+^CD27^+^ memory B cells were both significantly higher in MG patients than in HCs (CD19^+^ B cells: HCs: 0.89 (0.36, 1.45), MG patients: 1.94 (1.018, 3.773), *p* < 0.01; CD19^+^CD27^+^ memory B cells: HCs: 0.27 (0.16, 1.57), MG: 2.25 (0.77, 4.42), *p* < 0.01) (Fig. 1A, B).

We classified the patients into the OMG group and the GMG group according to the muscle involved, and we found a greater increase in CD19^+^ CD27^+^ memory B cells in GMG patients than in OMG patients when compared to the level in healthy people (HCs: 1.41 (1.05, 2.64), OMG: 2.04 (1.34, 2.04), GMG: 2.74 (1.87 4.05), *p* = 0.002, *p* = 0.02, respectively). Patients were divided into OMG and GMG for analysis. The elevated TRAF6 expression in CD19^+^ B cells and CD19^+^CD27^+^ memory B cells in GMG patients (28 cases) was greater than that in the corresponding cells in OMG patients (14 cases) with acute aggravation (CD19^+^ B cells: OMG: 1.76 (0.83, 3.26), GMG: 3.23 (1.51, 6.70), *p* = 0.03; CD19^+^CD27^+^ memory B cells: OMG: 1.94 (0.68, 3.38), GMG: 3.23 (1.61, 6.45), *p* = 0.03). TRAF6 expression was with acute aggravation (28 cases) compared with GMG in MMS (34 cases) (CD19^+^ B cells: acute: 3.23 (1.51, 6.70), MMS: 1.85(0.63, 3.41), *p* = 0.009; CD19^+^CD27^+^ memory B cells: acute: 13.23 (1.62, 6.45), MMS: 2.06 (0.67, 3.52), *p* = 0.028). TRAF6 expression in CD19^+^ B cells and CD19^+^CD27^+^ memory B cells was low, and there was no significant difference between OMG (24 cases) and GMG (34 cases) in the MMS state; however, the expression levels were still higher than those in healthy subjects (Fig. 1).

**Fig. 2:**
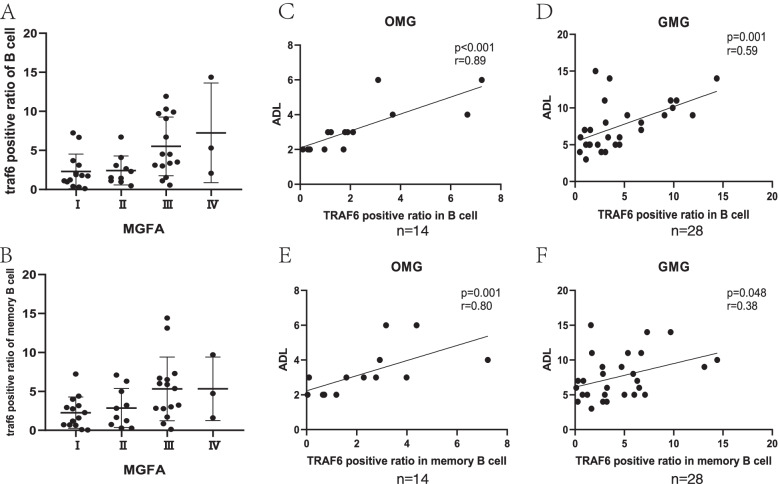
**A**, **B** MG patients with acute aggravation were classified according to the guidelines of the MG Foundation of America (MGFA). TRAF6 expression exhibited an increasing trend from type I to type IV, although there was no statistical difference. **C** Correlation between the TRAF6 expression level in CD19^+^ B cells and the ADL score in OMG patients with acute aggravation (*p* < 0.001, *r* = 0.89) **D** Correlation between the TRAF6 expression level in CD19^+^ B cells and the ADL score in GMG patients with acute aggravation (*p* = 0.001, *r* = 0.59). **E** Correlation between the TRAF6 expression level in CD19^+^CD27^+^ memory B cells and the ADL score in OMG patients with acute aggravation (*p* = 0.001, *r* = 0.80). **F** Correlation between the TRAF6 expression level in CD19^+^CD27^+^ memory B cells and the ADL score in GMG patients with acute aggravation (*p* = 0.048, *r* = 0.38)

Furthermore, the MG patients with acute aggravation were classified by the guidelines of the MG Foundation of America (MGFA). TRAF6 expression exhibited an increasing trend from type I to type IV, although there was no statistical difference. (Fig. 2A, B).

In our study, the positive rate of TRAF6 had no significant correlations with sex (Female:2.71(1.49, 4.18), Male:1.8(0.97, 4.10), *p* > 0.05), age or onset age (EOMG: 2.71(1.07, 4.18), 1.97(1.18, 4.10), *p* > 0.05).

Among the 89 patients, 22 patients were associated with thymoma, and 20 of them had undergone thymoma resection. In addition, 8 of the 22 patients with thymoma achieved MMS after treatment. In this case, we obtained the result that thymoma had no effect on the expression of TRAF6 (Thymoma: 3.05(1.95, 3.87), Normal: 1.84(1.02, 4.36), *p* > 0.05).

### Relationship between TRAF6 expression and MG-ADL score

We found that there was a significantly positive correlation between the TRAF6 expression level in CD19^+^ B cells and the ADL score in both OMG patients and GMG patients with acute aggravation (OMG: *p* < 0.001, *r* = 0.89; GMG: *p* = 0.001, *r* = 0.59) (Fig. 2C, D). The TRAF6 expression level in CD19^+^CD27^+^ memory B cells was also positively correlated with the ADL score in OMG and GMG patients with acute aggravation (OMG: *p* = 0.001, *r* = 0.80; GMG: *p* = 0.048, *r* = 0.38) (Fig. 2E, F).

### Expression of TRAF6 before and after treatment

**Fig. 3:**
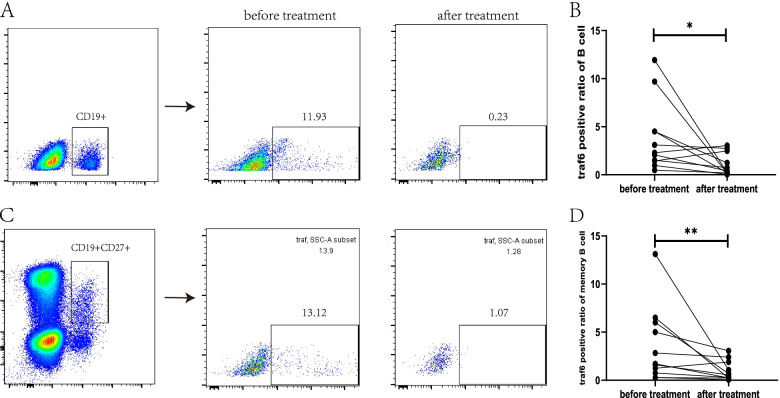
**A**, **B** Comparison of TRAF6 in CD19^+^ B cells collected from patients before and after treatment (before treatment: 2.30 (1.44, 4.52), after treatment: 0.40 (0.19, 2.48), *p* = 0.03). **C**, **D** Comparison of TRAF6 in CD19^+^CD27^+^ B cells collected from patients before and after treatment (before treatment: 1.71 (0.75, 6.00), after treatment: 0.56 (0.23, 3.06), *p* < 0.01)

In the 11 MG patients who were followed, TRAF6 expression was significantly decreased in both CD19^+^ B cells and CD19^+^CD27^+^ memory B cells after treatment (CD19^+^ B cells: before treatment: 2.30 (1.44, 4.52), after treatment: 0.40 (0.19, 2.48), *p* = 0.03; CD19^+^CD27^+^ B cells: before treatment: 1.71 (0.75, 6.00), after treatment: 0.56 (0.23, 3.06), *p* < 0.01) (Fig. 3).

## Discussion

In MG patients with acute aggravation, we observed that the TRAF6 levels in CD19^+^ B cells were upregulated in GMG compared with OMG, suggesting that the TRAF6 levels in CD19^+^ B cells are relevant to MG phenotypes and may be a useful biomarker of disease subtype. Additionally, a strong relationship between the TRAF6 expression in B cells and ADL score was identified, indicating that the TRAF6 expression level in CD19^+^ B cells was closely related to the severity of MG and might be an indicator for predicting disease severity. TRAF6 expression exhibited an increasing trend from type I to type IV, although there was no statistical difference, this may be due to the small sample, especially the type IV group with only three patients. More samples are needed in the further study. In addition, TRAF6 expression was significantly elevated in CD19^+^ B cells and CD19^+^CD27^+^ memory B cells in GMG with acute aggravation compared with GMG in MMS.

To date, the role of TRAF6 in MG has been unclear. However, a role for TRAF6 expression as a proinflammatory marker has been demonstrated in several autoimmune diseases, such as SLE [8] and RA [10]. Suzanne et al. reported that the CD40-TRAF6 signaling pathway plays a key role in neuroinflammation and demyelination in experimental autoimmune encephalomyelitis (EAE) [14]. Wu et al. demonstrated that NUR77 affects the development of inflammatory bowel disease (IBD) through the TRAF6/TIR-IL-1R signaling axis [15]. Previous studies have shown that the NF-κB, MAPK, Akt (also known as protein kinase B) and interferon regulatory factor pathways, which are involved in abnormal B cell proliferation and excessive antibody production, contribute to the pathogenesis of MG and that TRAF6 is the upstream intersection of these pathways [16–20]. In addition, the inflammatory cytokines produced by these pathways, such as IL-6, tumor necrosis factor-α (TNF-α), and interferon-γ (IFN-γ), were found to be involved in the pathogenesis of MG. Moreover, these cytokines were shown to be produced by B cells [21–23]. Thus, we speculated that elevated TRAF6 expression in CD19 + B cells is involved in the pathogenesis of MG through the CD40-TRAF6 signaling pathway through which it regulates the activation of the NF-κB and MAPK signaling pathways.

Several studies have demonstrated that memory B cell numbers are significantly increased in MG patients compared with healthy people [24, 25]. A study showed a significant increase in CD19^+^CD27^+^ B cells but not total CD19^+^ cells at relapse in rituximab-treated MG patients [26], indicating that memory B cells predict acute relapse. Consistent with previous studies, our results revealed a higher frequency of CD19^+^CD27^+^ B cells in GMG patients than in OMG patients or HCs. On the other hand, our results identified that the TRAF6 levels in CD19^+^CD27^+^ memory B cells were upregulated in GMG patients compared with OMG patients in the acute aggravation state and that there was a positive relationship between the TRAF6 level in the memory B cells of MG patients and MG-ADL scores, suggesting that overexpression of TRAF6 in memory B cells may predict disease severity in MG. TRAF6 contributed to the pathogenesis of MG, possibly by activating memory B cells, which are capable of long-term survival and rapid differentiation into antibody-secreting cells upon antigen re-encounter [27, 28].

In the 11 patients who were followed, the TRAF6 expression in CD19^+^ B cells and CD19^+^CD27^+^ memory B cells was significantly decreased in the MMS achieved after treatment. Our study is limited by the comparation of MG patients before and after immunotherapy in one center. In addition, the sample size of the study was small. Whether the effect of immunosuppressive therapy on TRAF6 is directly related to MG regression will require further study. A study on human renal proximal tubular epithelial cells showed that inhibition of the MyD88/TAK1/TRAF6/IRAK1 complex significantly attenuated the cytotoxicity of proinflammatory mediators and suppressed their release [29]. Two other observational studies have demonstrated that the CD40-TRAF6 signaling pathway plays key roles in neuroinflammation and demyelination in EAE and that inhibition of the CD40-TRAF6 pathway slightly reduces the severity of EAE [14, 30]. Given that reduced TRAF6 expression levels are correlated with improvement in various autoimmune disease models and our results showed a strong relationship between TRAF6 in B cells and disease severity, TRAF6 might be a potential therapeutic target in MG.

## Conclusion

We observed upregulated expression of TRAF6 in CD19^+^ B cells and CD19^+^CD27^+^ memory B cells, which was significantly associated with the disease severity of MG, and a significant decrease in TRAF6 after immunotherapy. Therefore, TRAF6 is potentially a useful biomarker of inflammation in patients with MG, and TRAF6 might be used to evaluate the effectiveness of treatment.

## Supplementary Information

**Additional file 1. **Data and information of Healthy Control. Data and information of MG patients with acute aggravation. Data and information of MG patients with MMS. Before and after treatment data of MG patients followed up.

## Data Availability

The datasets used and/or analyzed during the current study are available in the supplementary document.

## Abbreviations

TRAF6: Tumor necrosis factor receptor-associated factor 6
MG: Myasthenia gravis
ADL: Activities of daily living
AChR: Acetylcholine receptor
MuSK: Muscle-specific kinase
LRP4: Lipoprotein-receptor-related protein 4
TLR: Toll-like receptor
NF-κB: Nuclear factor-kappaB
MAPK: Mitogen-activated protein kinase
IL-6: Interleukin-6
IL-4: Interleukin-4
IL-1: Interleukin-1
SLE: Systemic lupus erythematosus
RA: Rheumatoid arthritis
PBMCs: Peripheral blood mononuclear cells
HCs: Healthy controls
MMS: Minimal presentation state
IFN-γ: Interferon-γ
EAE: Experimental autoimmune encephalomyelitis
BCRs: B cell antigen receptors
LMP1: Latent membrane protein 1
IRAK1: IL-1 receptor-associated kinase 1
